# Sample-centred shimming enables independent parallel NMR detection

**DOI:** 10.1038/s41598-022-17694-y

**Published:** 2022-08-19

**Authors:** Yen-Tse Cheng, Mazin Jouda, Jan Korvink

**Affiliations:** Institute of Microstructure Technology (IMT), Karlsruhe Institute of Technology (KIT), 76344 Eggenstein-Leopoldshafen, Germany

**Keywords:** Solution-state NMR, Electrical and electronic engineering

## Abstract

Two major technical challenges facing parallel nuclear magnetic resonance (NMR) spectroscopy, at the onset, include the need to achieve exceptional $B_0$ homogeneity, and good inter-detector radiofrequency signal decoupling, and have remained as technical obstacles that limit high throughput compound screening via NMR. In this contribution, we consider a compact detector system, consisting of two NMR ‘unit cell’ resonators that implement parallel $B_0$ shimming with parallel radiofrequency detection, as a prototype NMR environment, pointing the way towards achieving accelerated NMR analysis. The utility of our approach is established by achieving local field correction within the bore of a 1.05T permanent magnet MRI. Our forerunner platform suppresses signal cross-coupling in the range of $-21$ dB to $-30$ dB, under a geometrically decoupled scheme, leading to a halving of the necessary inter-coil separation. In this permanent magnet environment, two decoupled parallel NMR detector sites simultaneously achieve narrow spectral linewidth, overcoming the spatial inhomogeneity of the magnet from 400 to 28 Hz.

## Introduction

Magnetic resonance spectroscopy (MRS) enables the robust characterization of organic and inorganic chemical samples whose spin populations are aligned by a strong and uniform $B_0$ magnetic field. The evolution of the spin population state is initiated by a radiofrequency excitation pulse, and after a period of manipulation and further evolution, its time-dependent response is recorded at high resolution^1–5^. Conventional NMR is primarily conducted in a time-inefficient sequence that can include one or more of the following steps: sample loading, coil tuning and matching, $B_0$ field shimming to an acceptable $B_0$ distortion level, and radiofrequency (RF) excitation and reception at the target NMR frequencies, and long recovery time further restrict the speed of acquisition. Altogether the operations are time-consuming and reduce sample throughput.

Current approaches from a hardware perspective, to boost up experimental speed and address the low throughput problem in current magnet systems, include automated sample loading^6–8^, multinuclear RF detectors^9–11^, and automated tuning and matching systems^12,13^. For instance, an automated flow-through system, integrated with liquid sample sensing, has proven that a large batch of sequential fluidic samples can be handled, each within 2.4 s, with sufficiently high NMR resolution^7^. Broadband detectors were utilized to simultaneously acquire multinuclear NMR spectra without further tuning and matching, or switching of coil topology^11^. Despite these significant technical advances in high throughput NMR, the cumbersome procedure of a typical sequential measurement still limits current NMR measurements, which motivates us to explore parallel spectroscopy.

Inspired by the idea of the parallel MRI array^14–18^, we aim to perform NMR spectroscopy in a microcoil array^19,20^. True parallelization of detection would dramatically reduce acquisition time but would require a dense array of decoupled RF detection coils^16,17^ The phased array imaging coil is a successful multiple-detector technology^16,17,20,21^ that employs coil overlap, embedded preamplification, and spatial orthogonality of the detected signal distributions. This promotes decoupling and hence good spatial localization of the spin signals, yet the technique does not decouple sufficiently for spectroscopy, which is more demanding in terms of sensitivity to stray signals. Moreover, even though coupling can be suppressed in such technique, the sample is inevitably detected by two adjacent coils due to the overlap topology. Three main constraints currently limit the application of multiple closely spaced coils in NMR spectroscopy.

1. *Inter-coil inductive coupling* Signal coupling among nearby coils, specifically due to the injection of electromagnetic energy from one coil to another, results from the Faraday induction and capacitive coupling among elements of the array. As described by Faradays’ law of induction, the electromotive force $\varepsilon _{i}$ in loop *i*, is defined as the rate of magnetic flux $\Phi _{ij}$ passing through the enclosed area of inductor loop *i*, as caused by a current in inductor loop *j*:
  1 $$\begin{aligned} \varepsilon _{i}=-N_{j}\cdot \frac{d\Phi _{ij}}{dt}=\frac{d}{dt}\iint _{\text {loop i}}^{}{\varvec{B}_{j}}\cdot d{\varvec{A}_i}. \end{aligned}$$
  The magnetic flux seen by the sample in loop *i*, in turn, depends on the magnetic flux density from the neighbouring loop ${\varvec{B}_{j}}$, as well as the loop’s own enclosed area. Hence this causes the recorded spectra to contain signals from both neighbouring samples. On the other hand, a voltage drop in one loop may overlap with a neighbouring loop, either heating it, or hindering charge movement, so will also imprint an induced signal among neighbouring loops. Whereas these correlations among coils could be removed in principle, for example invoking orthogonality of the sensitivity map, and subsequent unravelling of coil signals, the current noise will also couple and cannot be removed, leaving a significant degradation of the signals since clean separation becomes difficult. As indicated in Wang et al.^22^, eight identical solenoid coils were introduced for parallel NMR using separation for decoupling, with 1 to 5% of the NMR signal bleeding among the coils.
2. *Shimming of multiple neighbouring samples* Sufficient homogeneity of the $B_0$ field ensures NMR accuracy, particularly in terms of spectral resolution, and for achieving sufficient signal-to-noise ratio (SNR) for detection. However, the magnetic field inside a magnet is inevitably compromised by imperfections of system components. These include the coil windings, strain caused by temperature differences, and the assembly of components from a variety of materials whose magnetic susceptibility properties cause local field variations, and hence tend to broaden the resulting spectrum^23,24^. The operation of a regular shim set in the magnet nevertheless is not able to compensate for the imperfections caused by a dense detector array, since the array’s orthogonal decomposition would require excessively high orders of compensation coils.
3. *Adjacent pulsed field gradient coils* Many important spectroscopic tasks rely on the availability of strong, short gradient pulses. Especially in screening experiments, pulsed field gradients are used for a variety of tasks, from temporary suppression of solvent signals, to accessing diffusion parameters, and so would be desirable within an arrayed detector system. Gradient coils can be constructed to be shielded, so that their field modification is only experienced in a volume of interest, but typically require a double layer coil that is less efficient (i.e., generates more waste heat per current used), but as a benefit, suffer less from eddy current effects. Sufficient gradient shielding also reduces externally caused magnetic forces on the gradient coil, in which the field of one gradient interacts with the current in another gradient coil. This topic is currently not discussed in the literature.

Previously proposed approaches^22,25^ for addressing the $B_0$ homogeneity of a radiofrequency coil array is through the filling of intermediate space with a susceptibility matching material to remove local field distortions at the interface between a coil and its surroundings, or the utilization of multiple miniaturized coils. However, these approaches are limited: (1) Susceptibility variations among samples are not addressed. And it’s nearly impossible to shim multiple samples to an optimal linewidth due to the position-varying susceptibility jump. As indicated in the article^22^, linewideth is broadened from optimal value (2–4)–(3–6) Hz. (2) For microcoils, the sample volume is also limited, which affects the signal-to-noise ratio. Another method is motional averaging^26^, achieved by rotating the sample along one axis to up to 60 Hz. However, an additional DC motor and gearbox is not favored in a high field magnet environment for two reasons: (1) DC motors are usually equipped with permanent magnets, or high susceptibility materials, and non-magnetic motors rarely have enough power and compactness; (2) The extensional gearbox system makes the resulting probehead design complicated, since space is quite limited in typical magnet bores.

Current technologies for decoupling adjacent coils include induced current compensation^27^, capacitive and inductive decoupling networks^28,29^, self decoupled coils^30^, all of which have proven to provide an acceptable decoupling value of ($<{-20}\,\hbox {dB}$). However, the methods target planar surface coil arrays that allow either capacitors to be attached to the RF coil, or additional loops used to achieve coil overlapping structures^21^. These methods are not favored for spectroscopy for numerous reasons: (1) Surface coil arrays target the study of live animal and human subjects, and the detector size allows bulky capacitors to be attached, which limit the potential of miniaturization; (2) The topology of a surface coil is not ideal for spectroscopy, mainly in terms of $B_1$ field homogeneity; (3) Surface coils are usually single-looped, which limits their sensitivity.

Therefore, considering the constraints of previous methods, we here explore the idea of localized spherical harmonic (SH) shim coils for a parallel detector system. Hsu et al.^31^ introduced a concept to locally homogenize the magnetic field in the interior portion of the frontal lobe, where the field is most seriously distorted, by placing resistive shim coils in the patient’s mouth. The advantage of their design lies in the ability to adjust the shim current, thus being able to create a strong correcting field. However, the shim order is limited. In our concept, localized shimming could achieve two advantages: (1) Allow simultaneous $B_0$ field correction at multiple positions; (2) Introduce the ability to correct strong field inhomogeneities, since the shim coils would be placed closer to the sample; (3) Introduce a cylindrical design, which would enable implementing multiple order spherical harmonic shims. For the suppression of signal coupling, we explored a geometrical decoupling technique, without interfacing with the RF coil, in order to mitigate spectral coupling.

### NMR cells in parallel

Commercial NMR magnet systems are typically designed such that they have a single RF coil, and more significantly, a single shim set, thus allowing the measurement of a single sample at a time. Shim systems in commercial magnets are based on the concept of co-located spherical harmonics, and therefore their functions are guaranteed orthogonal only relative to a single coordinate origin, and only to some extent, since neither the sample nor coil are ideally spherical. Herein, we propose a parallel NMR system for 1.05T field magnet, capable of spatial field correction enabling individual and simultaneous NMR detection.

**Figure 1:**
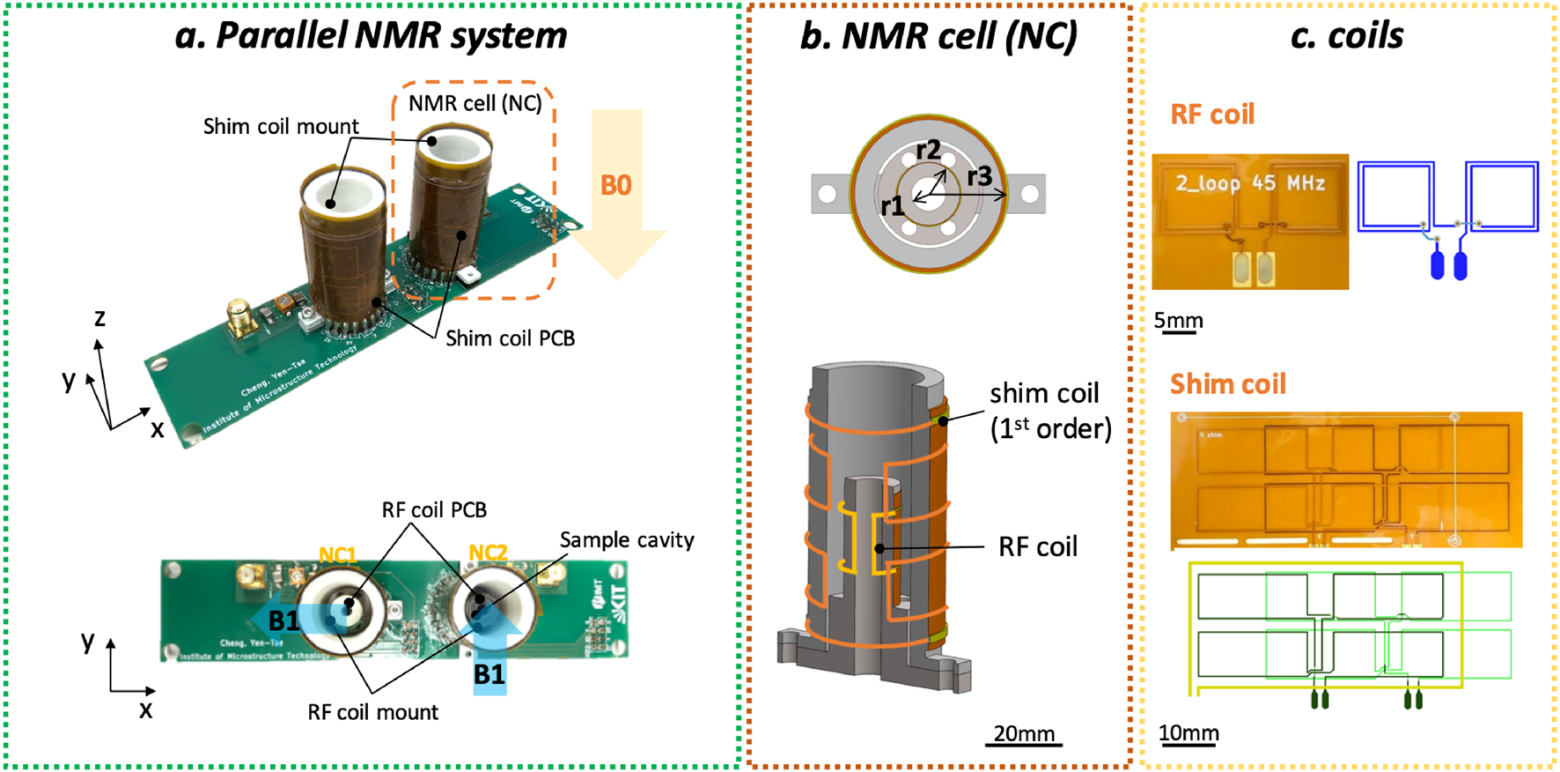
(**a**) Photograph of the proposed parallel system, featuring two NCs mounted on a PCB. (**b**) CAD model of a single NC, featuring a 3D printed holder for the RF signal detection coil and a circular capillary, and a set of miniaturized shim coils surrounding the detection site ($r_1={2.5}\,\hbox {mm}$, $r_2={4}\,\hbox {mm}$, $r_3={8.5}\,\hbox {mm}$.) (**c**) Photograph and schematic of a flexible substrate RF coil PCB, and a shim coil stack PCB, prior to wrapping the PCBs onto the support.

Figure 1a demonstrates a parallel NMR system built from a highly integrated NMR cell (NC) as a standalone NMR sensing unit, and conservatively expands the number of units to achieve moderate parallelism. Each NC is composed of an RF coil, for NMR signal excitation and reception, and a set of local shim coils for spatial static field correction. The directions of the $B_1$ vectors generated by each NC are orthogonal to each other to mitigate magnetic flux coupling. The schematic of the unit NC is shown in Fig. 1b. Both shim coil sets and RF coils are made using flexible printed circuit board technology (Fig. 1c) and are rolled up around 3D-printed PLA-based supports. The sample holder is made for an $r_1={2.5}\,\hbox {mm}$ radius glass tube that is used as a sample holder. A saddle coil with a radius of $r_2={4}\,\hbox {mm}$ was utilized as the RF NMR detector. The shim coils were wrapped around the outer support which has a radius of $r_3={8.5}\,\hbox {mm}$. The RF coil is based on the Ginsberg et al.^32^, presenting the optimal geometry of saddle coil for producing a uniform magnetic field. Details on the design of the NC shim can be found in the Supplementary Materials.

The localization of the shim coil sets thus divides the regular isocenter of the magnet into multiple, sample-defined isocenters, allowing for local and separate field correction. The technical advantage of a local shim system mainly lies in the independent operation of these orthogonal spherical harmonic coil fields when constructing the desired correction profile.

## Results

We have designed and implemented an NMR probehead incorporating the two single channel NCs, each resonant at a Larmor frequency of 44.93 MHz, for operation in a 1.05T permanent magnet MRI system. This subsection will demonstrate the results of parallelised shimming, and the acquisition of separate spectra utilizing the prototype.

### Geometrical consideration of misaligned-decoupled coils

**Figure 2:**
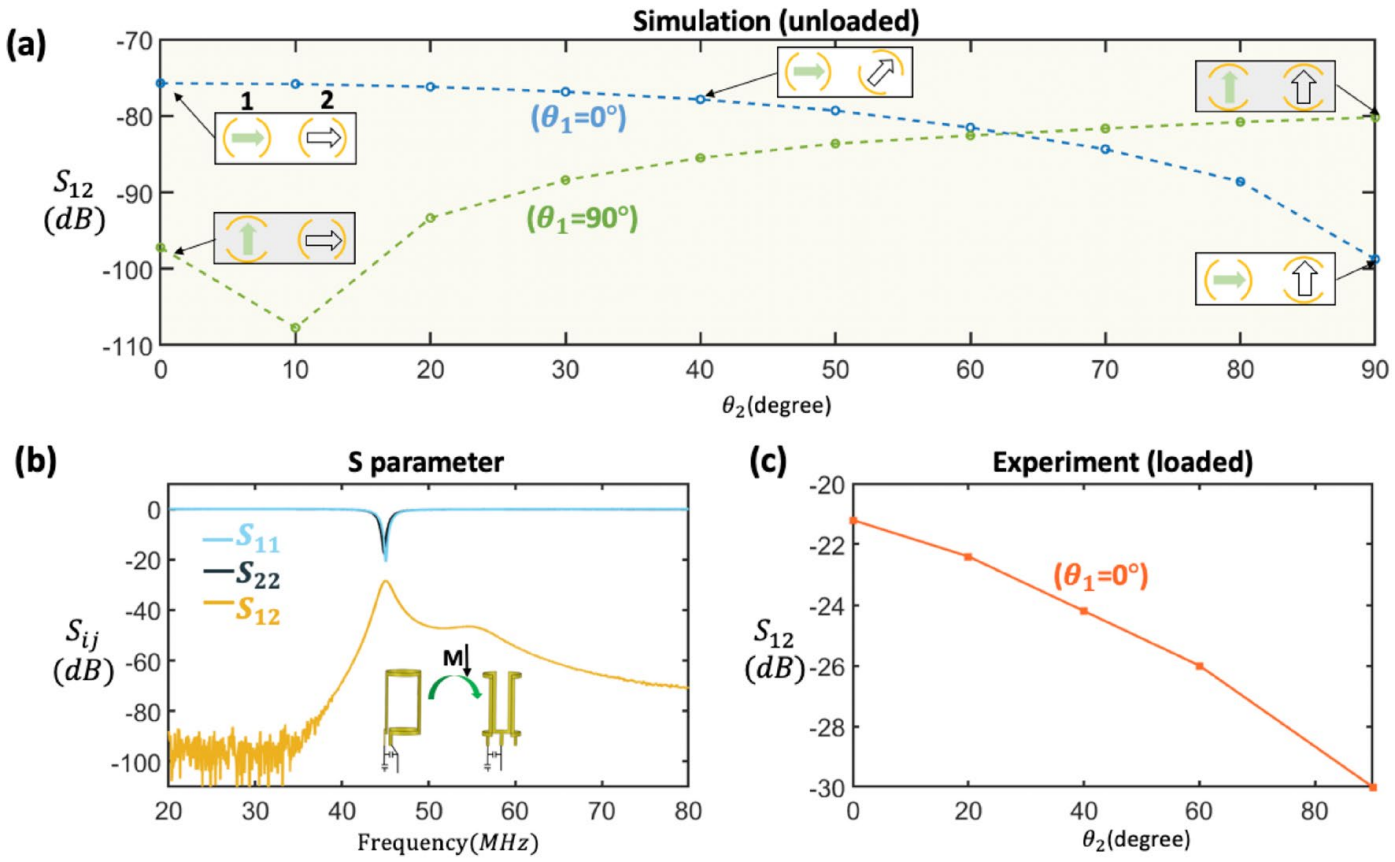
(**a**) Simulated coupling coefficients for two saddle coils at 45 MHz, for a range of misalignment angles obtained by rotating NC2 (Blue: Coil NC1 is fixed at ${0}^{\circ }$. Green: Coil NC1 is fixed at ${90}^{\circ }$). (**b**) Measured scattering parameters $S_{11}$, $S_{22}$, and $S_{12}$ versus frequency for NC1 and NC2, while employing the proposed geometrical decoupled technique. Both NCs are tuned to 45 MHz and matched to ${50}\Omega$. (**c**) Loaded coupling coefficients $S_{12}$ measured between NC1 and NC2 for different misalignment angles. Coil NC1 is fixed at ${0}^{\circ }$.

To evaluate how the geometrical misalignment of two adjacent unloaded coils contribute to the mitigation of cross coupling, we simulated and measured the scattering parameter set $S_{12}$ for different angles of rotation ($\theta _1$ and $\theta _2$) of each coil (NC1 and NC2) respectively. Based on the Biot-Savart law:

2 $$\begin{aligned} \varvec{B}\big (\varvec{r}\big ) = \int \frac{Id\varvec{l} \times \varvec{r}'}{ \vert \varvec{r}' \vert ^{3} }\frac{\mu _{0}}{4\pi } \end{aligned}$$

and Faraday’s law of induction (1), as the coil-to-coil distance $\mathbf {r}^\prime$ is decreased, the coupling will increase significantly. We fixed the inter-coil separation to 35mm, so that the result will only take the $B_1$-misalignment into account. The simulation result is shown in Fig. 2a, indicating a reduction of $S_{12}$ to − 100 dB when ${0}^{\circ }\le \theta _2\le {90}^{\circ }$ is varied while $\theta _1$ is fixed at ${0}^{\circ }$ (blue curve).

Setting $\theta _1$ to ${90}^{\circ }$, and again varying ${0}^{\circ }\le \theta _2\le {90}^{\circ }$, we see that $S_{12}$ reaches a maximum of ${-80}\,\hbox {dB}$ (green curve). A dip at ${10}^{\circ }$ is contributed by the slightly imperfect magnetic dipole field of the saddle geometry. The result indicates a strong suppression of signal coupling (23 dB) owing to the integrated projection of RF field of coil 1 onto the sensitive volume of coil 2 induces the minimal current when both coils field vector are orthogonal. Although only valid for unloaded coils, orthogonal misalignment can nevertheless greatly reduce the magnetic flux coupling also when coils are loaded with tank circuit. We thus fixed the relative orientation to ${90}^{\circ }$, tuned both resonators to 45 MHz, and matched them to ${50}\Omega$, obtaining less than ${-20}$ dB of reflected energy ($S_{11}$, $S_{22}$) and ${-30}$ dB of coupling ($S_{12}$) using a network analyzer, and presented in Fig. 2b. For loaded coils, a plot of the measured $S_{12}$-parameters as a function of $\theta _2$ is shown in Fig. 2c. As expected, the parallel coils have higher coupling than for the misaligned case, with a transmission coefficient $S_{12}$ of ${-21.2}$ dB going down to almost ${-30}$ dB, as was found for the unloaded simulation result.

**Figure 3:**
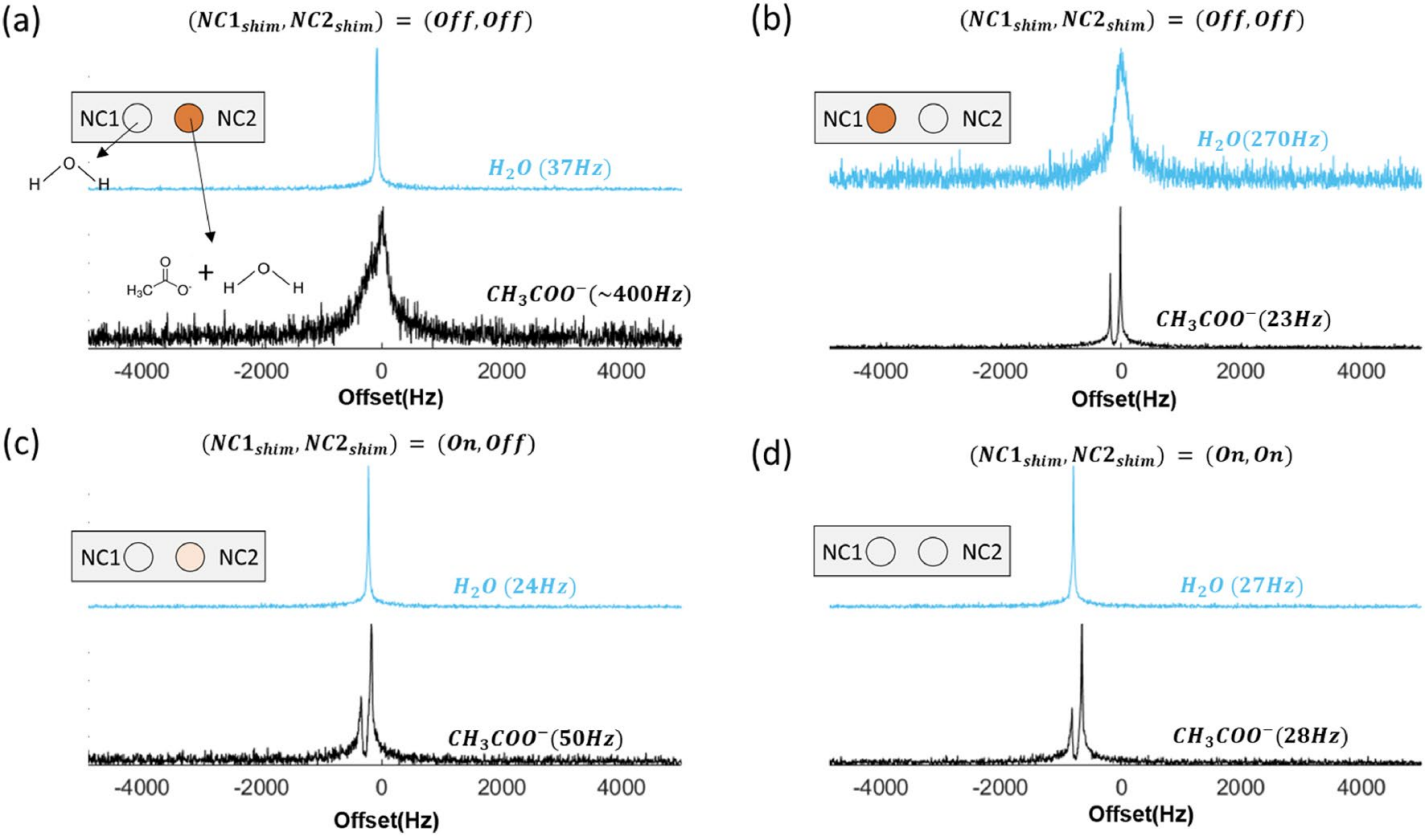
NMR experiment using the probehead to observe required shimming value and procedure for achieving shimming on two NCs. (**a**) Stage 1: regular shimming on NC1 with strength $(111,194,434)~\hbox {Hz cm}^{-1}$ (**b**) Stage 2: regular shimming on NC2 with strength $(-313,216,189)~\hbox {Hz cm}^{-1}$. (**c**) Stage 3: use the intermediate regular shimming value $(-200,194,250)~\hbox {Hz cm}^{-1}$ and turn on local shim of NC1. (**d**) Stage 4: turn on both local shim. (Note that the vertical axis is arbitrary, and the color of the circle is used to indicate the inhomogeneity of the $B_0$ field: deep orange > light orange > light gray).

### Parallel shimming strategy

In order to find a proper shimming set which is compatible with the regular shim set of the 1.05T magnet (ICON, Bruker), a sample-centered shimming coil for each NC was designed as a truncated spherical harmonic set. Given the space provided at the isocenter of the magnet core, each shim coil’s topology was determined using a finite element solver (COMSOL), as described in the “Methods” section.

The regular shim coil is able to reduce general inhomogeneities to within the µT range. The diameter of the dedicated shim coil was chosen to be $d_3={17}\,\hbox {mm}$, to ensure its ability to remove any remaining inhomogeneity with reasonable current intensity. This in turn determined the separation among NCs, since shimming coil fields should only minimally interfere with each other, resulting in an inter-NC separation of 35mm. Thus each NC achieved a shimset coordinate origin within its own sample volume.

Figure 3 depicts the result of shimming operations for the dual NC probehead (Fig. 1a) employed using a series of single pulse experiments. For this experiment, NC1 contained pure distilled water, and NC2 contained 50wt.% actetic acid in diluted water. To perform parallel field shimming in practice, all three sets of shims were employed: the NC1 and NC2 shimsets surrounding the detection sites, and the regular shimset as supplied by the manufacturer of the magnet. For each of the sets, we only considered the first order terms (*X*, *Y*, *Z*) in correcting the spatial $B_0$ field variation. The quality of the $B_0$ homogeneity was determined from the acquired NMR spectrum by monitoring its full-width half-maxumum (FWHM). The shimming proceded as follows:

- *Stage 1* Based on the DI water sample in NC1, the regular shim settings were determined to have an (*X*, *Y*, *Z*) strength of $(111,194,434)~\hbox {Hz cm}^{-1}$ to achieve a FWHM linewidth of 37 Hz. For these settings, NC2 delivered a noisy and broad spectrum, with a FWHM of 400 Hz, in which the Methyl group of the acetate sample was not discernible.
- *Stage 2* The regular shim settings for NC2 were found to have an (*X*, *Y*, *Z*) strength of $(-313,216,189)$, to achieve a FWHM linewidth of 23 Hz, acquired on a $^1$H peak of acetate, yet to have a FWHM linewidth of 270 Hz for the $^1$H peak of the DI water sample in NC1.

We then linearly interpolated the global X and Z shim value between its settings for stage 1 and 2, i.e., obtaining an (*X*, *Y*, *Z*) strength of $(-200,194,250)~\hbox {Hz cm}^{-1}$, which represented a reasonable compromise for the action of the global shim over the entire sensitive volume. We then continued as follows:

- *Stage 3* As shown in Fig. 3c, the local NC1 shim was now turned on with a local coilset current of $(X,Y,Z)=(46.6, 0.0, 97.8)\,\hbox {mA}$, whilst retaining the interpolated regular shim setting of $(-200,194,250)~\hbox {Hz cm}^{-1}$ This achieved 24 Hz of FWHM on the $^1$H peak, whereas for NC2, a FWHM linewidth of 50 Hz was obtained without applying any current to its shimset.
- *Stage 4* In a final step, the NC2 shim was also turned on, with an (*X*, *Y*, *Z*) shimset current of $(2.9, 0.7, 23.6)\,\hbox {mA}$, which for NC2 achieved a FWHM linewidth reduction to ${28}\,\hbox {Hz}$, but without affecting the narrowed linewidth of NC1.

**Figure 4:**
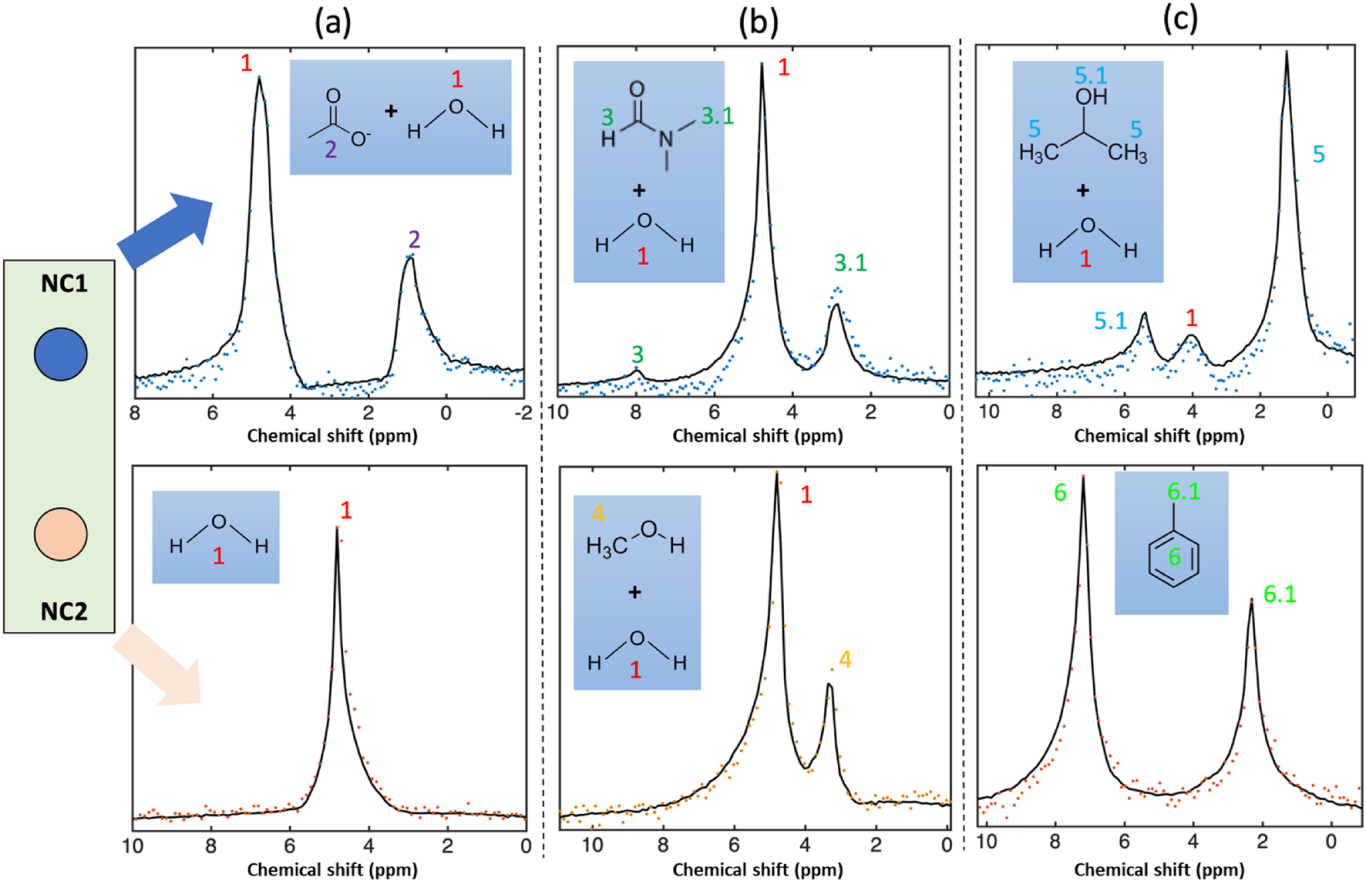
Measured $^1$H NMR spectra with synchronized excitation and reception (2 samples in 2 NCs) (**a**) $^1$H spectra of acetic acid aqueous solution (top) and DI water (bottom). (**b**) $^1$H spectra of N,N-dimethylformide (DMF) aqueous solution (top) and methanol in DI water (bottom). (**c**) $^1$H spectra of 2-propanol in DI water (top) and pure toluene (bottom). Peak numbering: $^1$H $\rightarrow 1$, acetic acid $\rightarrow 2$, DMF $\rightarrow \{3, 3.1\}$, methanol $\rightarrow 4$, 2-propanol $\rightarrow \{5, 5.1\}$, toluene $\rightarrow$ {6, 6.1}. (Note that the vertical axis is arbitrary.)

### Real-time NMR parallel spectrum

The NC based parallel system, as depicted in Fig. 1a, with characteristics shown in Fig. 2b, and the synchronized excitation and reception setup given in the “Methods” section, was assigned to detect $^1$H nuclei in parallel. Thus, six different samples, arranged into three sets of experiments (each set considered two distinct samples), were investigated. The aqueous samples utilized in our NMR characterization are not only common solvents that are widely used for biological processing, but also contain distinguishable peaks that help to reveal any cross coupling effects. NMR experiments were carried out at 45 MHz, corresponding to the $^1$H frequency of a 1.05T magnet. The results are shown in Fig. 4. Each spectrum presents a single scan, and the result of signal averaging enhanced SNR.

The parallel NMR acquisition of two samples is demonstrated in Fig. 4a from measurements of 1D $^1$H spectra in samples contained in the two NC’s. For each sample, 32 sequential scans in 32 s were carried out to increase the SNR in order to distinguish the NMR spectral peaks. Since the magnet displayed a few Hz s^−1^ of drift, the resulting spectra were post-processed with phase correction and spectral peak alignment after each scan (a frequency lock channel was not available). According to the chemical structure presented, both samples included $\mathrm {H}_2\mathrm {O}$ which contributed to the water peak. We aligned the $\mathrm {H}_2\mathrm {O}$ peaks as reference at 4.8 ppm, so that the final spectrum resolved the $\mathrm {C}\mathrm {H}_3$ and $\mathrm {O}\mathrm {H}$ peaks at 0.9 ppm and 4.8 ppm with a FWHM of 22 Hz. However, the J-coupling on the $\mathrm {C}\mathrm {H}_3$ peak was not discernable owing to limitation in the hardware, including the level of magnetization, and the highest shimming order of the magnet system. Even though no detected $\mathrm {C}\mathrm {H}_3$ coupling signal was present in $\mathrm {H}_2\mathrm {O}$ spectrum, both RF coils were operated in synchronized excitation and reception mode, which underscored the decoupling capability of the proposed NC system.

In a second experiment, Fig. 4b shows the spectrum of a mixture of both dimethylformamide (DMF) and methanol, each in an aqueous solution. At both measurement sites, 62 scans in 62 s scan time was applied. The $^1$H water peak for both samples was recorded at 4.8 ppm, the spectrum of DMF is resolved, with $^1$H peaks at 2.93 ppm, 4.8 ppm, and 8.1 ppm, and the spectrum of methanol with $^1$H peaks at 4.8 ppm and 3.36 ppm.

Figure 4c presents the spectrum of iso-propanol (IPA) and toluene in 32 scans. The spectra of both samples are referenced at their $\mathrm {C}\mathrm {H}_3$ peaks. IPA was referenced at 1.2 ppm, the other two $\mathrm {O}\mathrm {H}$ groups were resolved at 4.04 ppm and 5.38 ppm. For toluene, the benzene group peak was at 7.1 ppm, and the peak of the $\mathrm {C}\mathrm {H}_3$ group at 2.3 ppm.

Based on the experimental results, it was found that the main peak of each sample could be discerned for the evaluation of cross coupling. Second, it was clear that all three sets of experiments demonstrated sufficient decoupling of the NC coil system, with unwanted coupling signals remaining undetected. Third, the spectral linewidth and hence $B_0$ homogeneity did not experience any significant change over multiple scans. Fourth, the two samples can be excited in parallel, and detected simultaneously, thereby halving the acquisition time of an equivalent serial experiment.

## Discussion

State-of-the-art high-throughput NMR spectroscopy reaches its speedup goal through continuous flow arrangements of sample droplets, and is embedded within an additional, automated flow system^6,7^. Nevertheless, such systems are inherently serial, so that only one sample can be only characterized at any time, leading to the need to develop coil array implementations for increased throughput. The present article proposes a prototype of a multiple-coil system, based on an integrated NMR cell (NC), towards parallel NMR spectroscopy that potentially extends current high throughput techniques. Here, some challenges have been carefully tackled, such as providing shimming on multiple samples, and achieving geometrical inter-coil inductive decoupling. Our proof-of-principle $^1$H spectrum results have demonstrated that two side-by-side NCs can achieve the same specifications as a single detector, including a low linewidth and high SNR, implying the future possibility of down-scaling parallel NMR with better resolution and increased SNR, for use also within high field strength superconducting magnets. The potential of integrated systems facilitates the implementation of higher order shim coils, such that the linewidth can be further reduced, and would render rapid compound screening in mixtures possible. Admittedly, when more shim coils are implemented, the shimming procedure would grow in complexity. Therefore, we are currently investigating a shimming strategy of finding the optimal goal for each shim set, towards designing an algorithm for parallel shimming in future high detector count arrays. In conjunction with artificial intelligence strategies, the use of deep regression for NMR shimming^33^ is a promising approach with which to accelerate the entire array shimming process. Potentially, the concept can be extended to a larger array of NCs, thereby enabling parallel TX/RX, which would divide the experimental time budget by a factor equal to the number of implemented NCs.

A common method to isolate two transmission line elements, is to position the elements far apart, which reduces the magnetic field coupling by the square of the separation distance. In addition, we demonstrated that the cross coupling of two RF saddle coils can be further reduced with a geometrical misalignment technique. The coupling effect has been evaluated in both simulations, and in synchronized NMR experiments, which indicated a good isolation of two unit NMR cells. Geometrical placement is found to additionally reduce coupling by 23 dB (unloaded) and 9 dB (loaded).

Moreover, the technique is not restricted to saddle-shaped coils, instead, any coil with a similar dipole field can benefit from it. Besides, the technique applies also to micro-coils, allowing a larger ratio of distance to coil size, and when combined with shimming structures, enhances each NC’s isolation.

## Methods

### Finite element method (FEM) simulations

**Figure 5:**
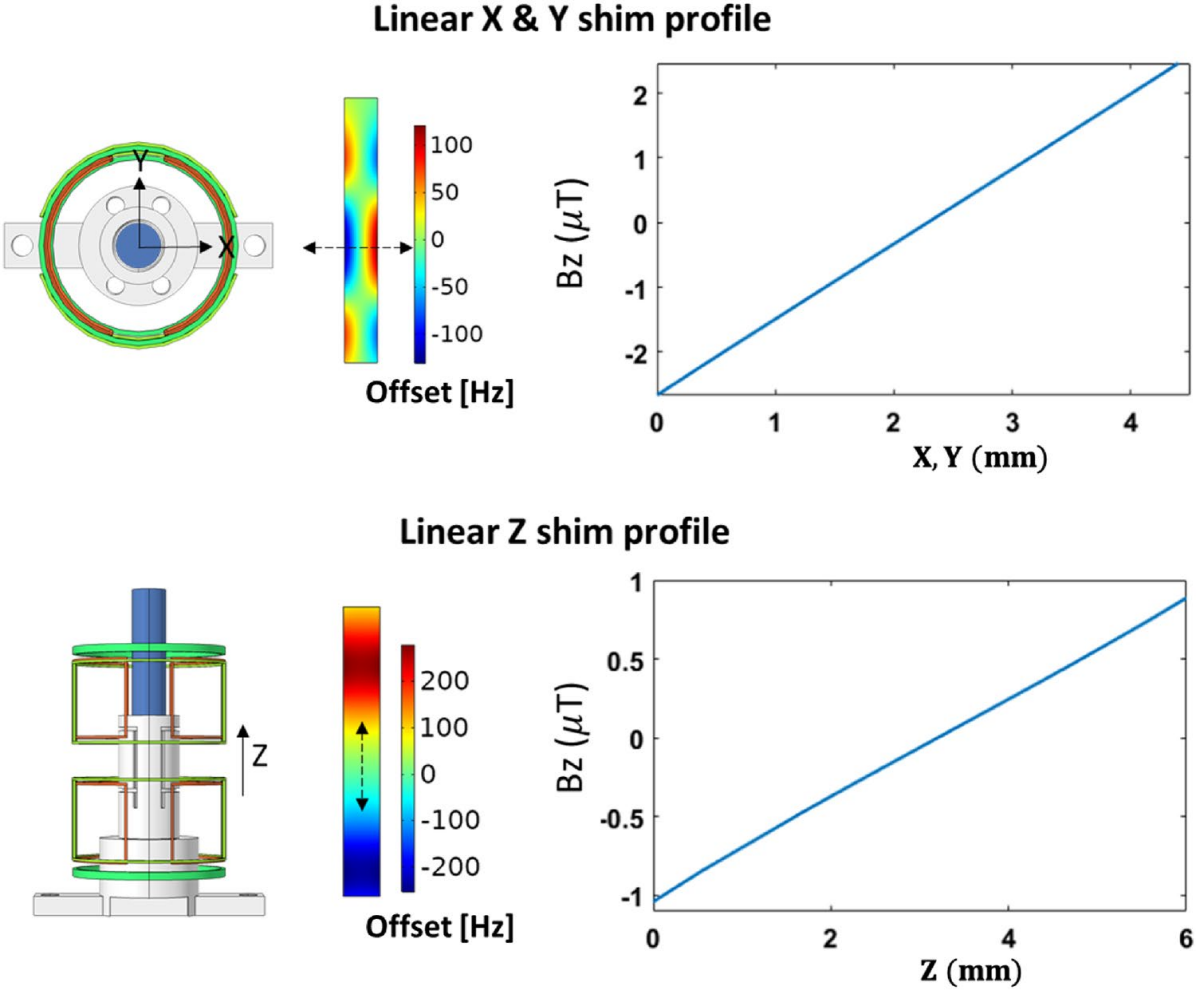
Finite element simulation result (COMSOL AB, Sweden). Linear shim field profiles over the sample region of interest of a generic NC, corresponding to the *X*, *Y*, and *Z* shim coils. The value of the current was preset to 100mA. Field values are given in µT, and dimensions in mm.

The shim and detector coil designs were based on finite element field computations using a commercial solver (COMSOL Multiphysics 5.4, AC/DC and RF modules, COMSOL AB, Sweden). The simulations yielded arrangements that reduced inter-coil coupling, and ensured proper field alignment. The topology of each shim coil was carefully designed considering the shimming capability and the spectral field-of-view. For the spatial variation present, the required strength required for shimming was found to be in the range of a few tens of µ TA^−1^, as seen from the shim values recorded in Fig. 3a. In the simulation, the value of the current was preset to 100mA. A polylactic acid (PLA) supporting structure was designed as a 3D-printable CAD file (SolidWorks, Dassault Systemes S.A.) and imported into COMSOL. The model and simulated shim profiles are present in Fig 5. The CAD model of the structure, of a similar diameter to mimic an NC, was flooded with water, with the three shim coils locally encircling the PLA. The entire NC was located inside a cylinder-shaped domain to model the surrounding air. Only homogeneous materials were specified. For radio-frequency simulations, performed to determine the effect of axial misalignment on inter-coil coupling, both coils were connected to uniform lumped ports with a characteristic impedance of ${50}\Omega$. A harmonic simulation was specified, with an excitation frequency of 45 MHz at the port of the first coil. Its alignment $\theta _1$ was maintained at both ${0}^{\circ }$ and ${90}^{\circ }$. For each of these settings, the second coil’s axial angle $\theta _1$ was swept from ${0}^{\circ }$ to ${90}^{\circ }$ in intervals of ${10}^{\circ }$, with the resulting $S_{12}$-parameter results plotted in Fig. 2a.

### Probe head manufacturing

The NC based probe head (depicted in Fig. 1a) including two NCs, bottom PCB, and PCB holder for connecting to the probehead of the ICON system, were manufactured as follows. The flexible PCB forming the shim and RF coils of the NCs, with overall thickness of 86mm, including 25mm polyimide substrate, two µm thick copper layers, and a 25 µm polyimide cover layer over the top copper layer, were ordered from a vendor (multiPCB, China). No additional surface finish was assigned, to prevent possible field distortions. Both coils were attached to supporting structures using epoxy (UHU Plus), and soldered to tracks on the bottom PCB. A PLA supporting structure was 3D printed in-house (Ultimaker 2+ 3D printer). The two completed NCs, arranged adjacently, were axially misaligned by ${90}^{\circ }$ to reduce $B_1$ field coupling, and attached by a nonmagnetic screw to the PCB through predefined holes.

### Materials

Samples for NMR characterization were prepared as follows: (1) 50% (v/v) aqueous solution of acetic acid; (2) Pure de-ionised water; (3) 33.3% (v/v) N,N-dimethylformide aqueous solution; (4) Solution of 40% (v/v) methanol in de-ionised water; (5) 50% (v/v) isopropanol aqueous solution; (6) Pure toluene. The solvents were ordered from a vendor (Sigma-Aldrich).

### Parallel NMR experiment setup

The parallel NMR experiment was carried out using a pre-clinical 1.05T cryogen-free MRI magnet system (Bruker Biospin, Ettlingen, Germany) together with a custom-built probe. The sample for each NC was prepared and inserted in the cavity as defined. During the synchronized NMR experiment, since the vendor-supplied ICON console is limited to single channel acquisition, the two NCs were each connected to one of two separate electronic interfaces, in parallel. One RF coil was connected to the ICON console and executed a single pulse experiment under the ParaVision software interface (version 6.0.1, Bruker). A second coil was connected to a low noise amplifier (ZX60-3018G-S+, Mini-Circuits ) for RX, a power amplifier (ZHL-20W-13SW+, Mini-Circuits) for TX, and the resulting signal was processed by a lock-in amplifier (UHFLI, Zurich Instruments), operated via the vendor-supplied software (LabOne 17.06, Zurich Instruments). To ensure parallel synchronization, a TTL transmission line of the ICON system was connected to the lock-in amplifier to provide a trigger signal for excitation. Thus both coils were excited simultaneously. For spectral averaging, each repetition was triggered by the ICON’s lock signal to ensure that, during each repetition, both coils were triggered at the same time. Multiple acquired spectra were post-processed, including peak shift adjustment and averaging.

## Supplementary Information

Supplementary Information.

## Data Availability

The raw data generated and analysed during the current study are available in the Cheng, Yen-Tse repository. https://doi.org/10.5445/IR/1000145508.
