# Supplementary material for: Sample-centred shimming enables independent parallel NMR detection
Source: Sci Rep. 2022 Aug 19;12:14149. doi: 10.1038/s41598-022-17694-y (PMC9389490; doi:10.1038/s41598-022-17694-y)
Supplement: Supplementary file 1 — Supplementary Information. [file 41598_2022_17694_MOESM1_ESM.pdf]

Supplementary Materials for Sample-centred shimming enables independent parallel NMR detection

Yen-Tse Cheng, Mazin Jouda*, and Jan G. Korvink*

*Corresponding author. Email: mazin.jouda@kit.edu, jan.korvink@kit.edu,

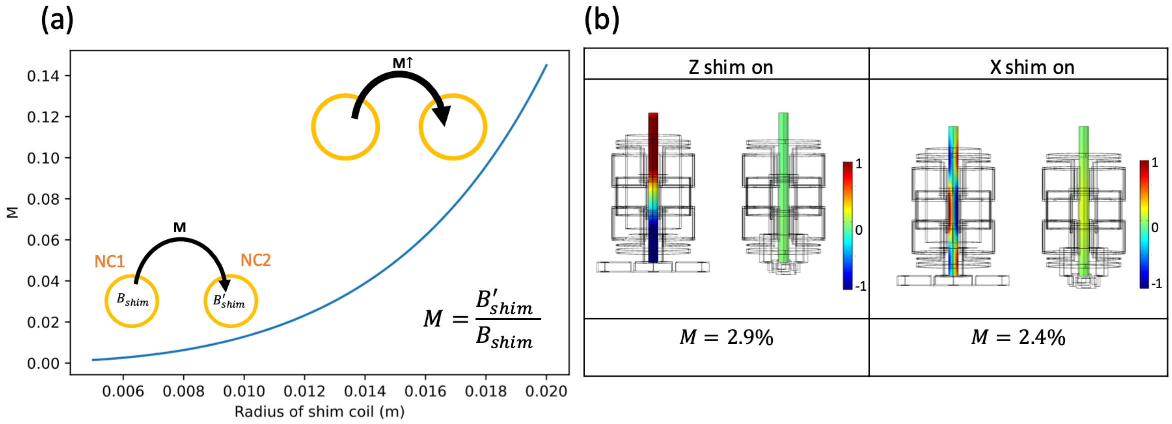

Figure S1: (a) Calculated stray field coupling between two circular loop coils with a range of radii based on Biot–Savart law. (M defines the static field coupling coefficient. B_{shim} is the maximum shimming field on the sample of NC1. $B^{'}_{shim}$ is the shimming field on the center of NC2.) (b)FEM simulation results of the stray field coupling from the shim coils (X and Z shims). The distance between two NCs is $35\,\mathrm{mm}$.

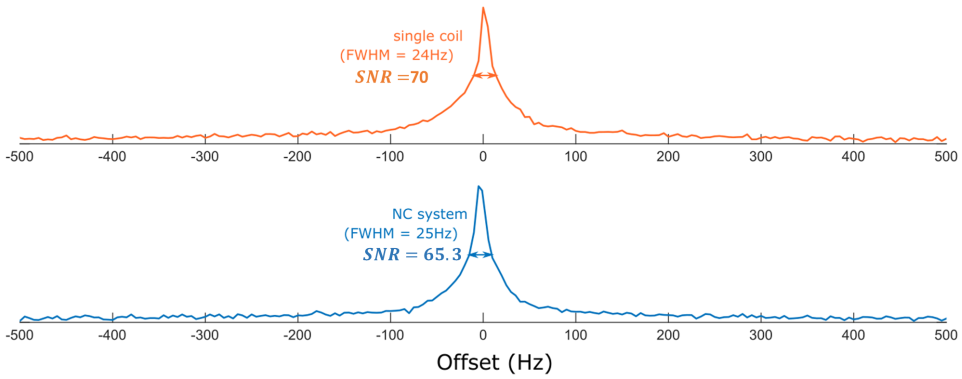

Figure S2: Measured ^{1}H NMR spectra with single coil system and dual-coil NC system. Both results are acquired at the optimal achievable line-width, $24\,\mathrm{Hz}$ for single coil system and $25\,\mathrm{Hz}$ for dual coil NC system.

Design of shim coil

We consider the stray field generated by the NC shim coil and the minimum shim capacity when designing a local shim coil for two NCs. Based on this, we proceed as follows:

- The homogeneous region (iso-center) of the magnet bore is $50\,\mathrm{mm}$(OD)x$40\,\mathrm{mm}$(L). Therefore, the distance between two NCs was set at $35\,\mathrm{mm}$ to reduce the stress of RF coupling.
- We measured the linewidth from two samples in two NCs to determine how strong the local shim field is required for two NCs. And, as shown in Figure 3b, the shim capacity of each NC shim must be greater than $400\,\mathrm{Hz\,cm^{-1}}$ to correct the spatial inhomogeneity.
- The topology design of the NC shim coil was based on the spherical harmonic correcting coils (x,y,z) as provided by Romeo et al.[1] on a cylindrical surface.
- The diameter of the NC shim coil is our design consideration: Because the shim is placed in very close proximity to the sample, a small diameter shim coil can provide a larger shim capacity. However, it will also generate a counter field, lowering the SNR of the RF coil. And unwanted noise would couple from the NC shims since they are not optimally shielded. When the diameter of the shim coil becomes too large, the NC1 shim will be close to NC2, and the stray field will be dominant.
- Therefore, We estimated the stray shim field coupling between two NC shims through Biot–Savart law and COMSOL multiphysics. The Biot–Savart law approximation is based on a circular current loop for a range of radii with a distance of 35mm inter-coil separation.
- Based on Biot–Savart law and COMSOL simulation, the stray field leaking in-between two NCs for a range of radii has been estimated, as shown in Fig. S1. Therefore, a reasonable value ($8.5\,\mathrm{mm}$) for the radius of shim was selected as it only allows around ($M = 2\,\%$ to $3\,\%$) stray field coupling for each shim. Nonetheless, the simulated result of linearity and shim capacity (in Fig. 5) shows the ability to remove the remaining inhomogeneity ($> 300\,\mathrm{Hz}$) with reasonable current intensity.

Single coil vs NC based dual coil system

To determine whether the implantation of a local shim with an RF coil causes noise or linewidth broadening, the traditional single coil spectrum is compared with the NC-based spectrum. Both are carried out under the same experimental conditions, which include the same RF coil, DI water sample, and excitation power. In the single coil test, only the regular shim is utilized. In the dual-coil test, regular shim, NC1 shim, and NC2 shim are utilized. This achieved $24\,\mathrm{Hz}$ of FWHM on the ^{1}H peak at the single coil system as shown in Fig.S2. For an NC based system, $25\,\mathrm{Hz}$ of FWHM is achieved, indicating an optimal linewidth can be achieved in the NC system. Nonetheless, the dual-coil NC system spectrum shows the similar SNR as to a conventional single coil system.

References

[1] Françoise Roméo and DI Hoult. “Magnet field profiling: analysis and correcting coil design”. In: *Magnetic Resonance in Medicine* 1.1 (1984), pp. 44–65.
